# A Survey of the Turkish Oncology Group (TOG): Are the Oncologists Trained for Decision Making on Geriatric Cancer Patients?

**DOI:** 10.7759/cureus.64533

**Published:** 2024-07-14

**Authors:** Sule Gul, Huseyin Tepetam, Omar Alomari, Didem Çolpan Öksüz, Serdar N Turhal, Fazilet Öner Dinçbaş

**Affiliations:** 1 Radiation Oncology, Health Sciences University, Dr. Lutfi Kirdar Kartal Training and Research Hospital, Istanbul, TUR; 2 Medicine and Surgery, Hamidiye International Faculty of Medicine, University of Health Sciences, Istanbul, TUR; 3 Radiation Oncology, Medical Faculty, Istanbul University-Cerrahpasa, Istanbul, TUR; 4 Internal Medicine, Department of Medical Oncology, Anatolian Health Center, Istanbul, TUR; 5 Radiation Oncology, Medical Faculty, Istanbul University-Cerrahpaşa, Istanbul, TUR

**Keywords:** medical-oncologist, radiation-ongologist, oncology, geriatrics, education

## Abstract

Introduction: Most newly diagnosed cancers occur in older adults, and it is important to understand a patient’s underlying health status when making treatment decisions. Therefore, clinicians need enhanced competencies and skills to effectively care for this population. However, most clinicians receive minimal to no training in geriatrics. This study aims to evaluate the education and training levels in geriatric oncology among Turkish physicians and to understand the factors influencing oncologists' treatment decisions for geriatric cancer patients.

Materials and methods: A 24-question survey was prepared to obtain the participating physicians’ demographic information, as well as to inquire whether they had received training during their medical education and residency on how to approach geriatric patients, in what process(es) they had received the training, whether they were currently treating geriatric patients, what they focused on when evaluating geriatric patients, and what they thought about their training and preparedness for approaching geriatric patients. The questionnaire was sent online to radiation and medical oncologists, and the link was published on the Turkish Society of Radiation Oncology and the Turkish Society of Medical Oncology websites.

Results: Two hundred and three physicians participated in the survey, 131 of whom were women. The median age was 41.66 years (24-69 years). One hundred and fifty-six physicians (76.1%) received specialty education at the university hospital. One hundred and three of them were radiation oncologists, and 80 were medical oncologists. Of the physicians, 19.7% received education in geriatrics before they specialized in oncology, and 6.9% said they were educated after specialization. When determining suitability for radiotherapy, 10.7% of radiation oncologists said that they use geriatric assessment tools. Similarly, 13.8% of medical oncologists claimed that they used geriatric assessment tools in determining suitability for chemotherapy. Of the physicians, 177 (86.3%) thought that geriatric evaluation could independently increase patient survival rate. Furthermore, patient cognitive status, functional status, physiological age, polypharmacy, geriatric specialist recommendations, inpatient services, patient relatives, and similar factors were found to be useful in treatment decisions. Finally, 92.7% of the participants believed that receiving education would have changed their perspectives on treating geriatric patients.

Conclusion: Our results provide perspectives on developing knowledge on and skills in geriatric training among oncologists. Learning new approaches is necessary for oncologists who more frequently confront geriatric patients with cancer.

## Introduction

As the population ages, the number of geriatric patients diagnosed with cancer also increases. According to the World Health Organization (WHO), while the onset of old age begins at 65, 65-74 years of age is considered young old age, 75-84 is considered advanced old age, and 85 years and older is considered very advanced old age. The number and proportion of people aged 60 years and older in the population are increasing. In 2019, one billion people worldwide were aged 60 years and older. WHO’s data indicate that this number will increase to 1.4 billion by 2030 and 2.1 billion by 2050 [1]. It has been predicted that by the 2050s, one in six people will be over 65 years old.

In North America, 70% of those with cancer and 50-60% of those newly diagnosed with cancer are 65 years of age and older. By 2030, new cancer cases in adults aged 65 and over are expected to increase by 67% [2-4]. According to Turkey’s 2018 population data, 8.8% of the population is aged 65 years and over [5].

Because aging itself is one of the biggest risk factors for cancer, the management of elderly patients has gained importance in the oncology field. Geriatric patients are biologically, functionally, psychologically, and socially different from younger patients. For this reason, approaches to geriatric patients require different perspectives. Moreover, the geriatric patient population is heterogeneous. It is important to identify geriatric patients with long life expectancies who may benefit from aggressive treatments. Thus, awareness of and education about geriatric oncology are important when selecting treatments and managing patients during and after treatment. However, in general, most oncologists do not know how to evaluate and manage geriatric patients. For example, even if a patient’s physiological conditions permit aggressive treatments, the patient might be left untreated because of their oncologist’s belief that treatment would not be tolerated. Therefore, oncologists need training to improve their knowledge bases and skills.

The need for further education in identifying the most appropriate treatment modalities for aging cancer patients was addressed by the American Society of Clinical Oncology (ASCO) in 1988 [4]. In their review, gaps in education and the need for continuing education were mentioned, and strategies to achieve success in this regard were suggested. The ASCO-Hartford Geriatric Oncology Scholarship, which deals with the education of physicians treating elderly oncology patients, is the largest and best-known educational initiative. Moreover, the International Society of Geriatric Oncology (SIOG) has worked to build a global database that includes training opportunities. One of SIOG’s top priorities is to integrate geriatric oncology education into the educational curricula of doctors and nurses working in geriatrics. Geriatric oncology topics were included in the ASCO curriculum in 2005 and in the European Society for Medical Oncology (ESMO)-ASCO curriculum in 2010, acknowledging the importance of educating medical oncologists in geriatric principles [6-10].

The increasing population of geriatric patients diagnosed with cancer has raised the question, “How much do we know about the approach to geriatric patients?” Besides monitoring for comorbidities and diminished performance status, geriatric patients should be evaluated more comprehensively, and personalized treatment planning should become standard. Therefore, on behalf of the Turkish Oncology Group’s Early-Late Side Effects Study Group, the aims of our study were to determine whether oncologists received training on geriatric patients, evaluate their approach to these patients, and identify factors that affect treatment decisions and management.

## Materials and methods

Study design, setting, and participants

This cross-sectional study aimed to assess the knowledge, attitude, and practice barriers towards geriatric patients among Turkish oncologists. The study was designed in accordance with the Strengthening the Reporting of Observational Studies in Epidemiology (STROBE) guidelines (Appendix) and aimed to compare the findings between the two demographics (radiation oncologists and medical oncologists). The inclusion criteria for the study were as follows: Turkish oncologists who have been working in Turkey at the time of data collection. There were no restrictions on sociodemographic factors. The study sought participation from eligible individuals by inviting them to complete a survey questionnaire. Convenience sampling was used to collect responses from the target population. 

Questionnaire and data collection

A comprehensive 24-question survey was meticulously developed to gather detailed demographic information from participating physicians. The survey sought to assess whether they had received training on how to approach geriatric patients during their medical education and residency training, the specific processes through which they had received this training, the proportion of these physicians actively treating geriatric patients, the factors they consider important when evaluating geriatric patients, and their overall perspectives on their training and preparedness in geriatric patient care. The survey was prepared and delivered to the physicians via the internet. This questionnaire was prepared in concordance with the study published by Morris et al. in 2017 investigating “Are Future Radiation Oncologists Equipped with the Knowledge to Manage Elderly Patients with Cancer?” and permission to use the questionnaire was obtained from the author [10]. In the questionnaire are seven descriptive questions (age, gender, geriatric education status, specialty, working year, geriatric education before/after specialty training, the institution where specialization training was received, the institution he/she worked for), six questions with 5-point scale (cognitive evaluation, getting geriatric expert support, comorbidity management, self-confidence in treatment), two evaluation questions with 7-point scale for geriatric patients (factors affecting radiotherapy dose selection, factors affecting chemotherapy dose selection), three questions for geriatric patients’ treatment recommendation, education with 5-point scale and six comment questions were used. The members of the Turkish Radiation Oncology Society and Turkish Medical Oncology Society contributed to the survey by utilizing the published survey link on their website.

For validation purposes, a pilot study of 20 participants was performed to ensure the clarity and reliability of the questionnaire. The Cronbach’s alpha of the scale was 0.65 which is an acceptable level of reliability. The feedback was taken into account and the questionnaire modified accordingly. The participants were invited to complete the questionnaire using Google Forms, and a link was sent via social media platforms. In addition, hard copies of the survey were also distributed to approach a greater number of participants. Data collection forms that were not filled out completely have been excluded from the analysis to ensure the accuracy and reliability of the results. Only fully completed forms were considered for inclusion in the study.

Ethical considerations

The study was performed in accordance with the Helsinki Declaration, and ethical approval was granted by the Institutional Ethical Review Committee of the Faculty of Medicine, University of Health Sciences, Turkey (2019/514/146/15). Participants were informed that their participation was voluntary and required informed consent, which was obtained by asking them to agree to the questionnaire on the first page of the online form.

Statistical analysis

All statistical analyses were conducted using SPSS version 20.0 (IBM Corp., Armonk, NY, USA). The participants’ demographics were summarized using descriptive statistics. The crossed chi-square test was used to compare classified categorical variables. Fisher Exact test was used in cases where the minimum expected value was less than 5, and Pearson Chi-square test was used in other cases. A p-value <0.05 was interpreted as statistically significant.

## Results

Two hundred and three physicians participated in the survey. One hundred twenty-three were radiation oncologists and 80 were medical oncologists. One hundred thirty-one of them were women (63.9%), and the median age was 41.6 (24-69) years old. The mean age of the radiation oncology physicians participating in the survey was 41.19, and 95 (77.2%) were women. The mean age of the surveyed medical oncology physicians was 42.39, of which 45 (56.3%) were male. One hundred fifty-six of them received their specialty education at a university hospital (76.1%). The number of physicians with less than 10 years of working experience was 96 (47.3%), and 107 (52.7%) had more than 10 years of working experience. Demographic information and the characteristics of the physicians are shown in Table 1. The most common primary tumor group of interest of the physicians was breast cancer (77.6%), followed by gastrointestinal system cancer (65.9%) and lung cancer (64.4%), respectively. 20.5% of the physicians defined age 65 as the elderly patient while only 1% of them stated that over the age of 85 was considered elderly patients.

**Table 1:** Demographic information and characteristics of the participants

Category	Radiation Oncologist N (%)	Medical Oncologist N (%)	Total N (%)
Gender			
Male	27 (13.3)	45 (22.2)	72 (35.5)
Female	96 (47.3)	35 (17.2)	131 (64.5)
Education received from			
University	94 (46.3)	64 (31.5)	158 (77.8)
Education hospital	29 (14.1)	16 (7.9)	45 (22.2)
Working years in the field			
1-10 years	44 (21.7)	52 (25.6)	96 (47.3)
10-20 years	59 (29.1)	15 (7.4)	74 (36.5)
Over 20 years	20 (9.9)	13 (6.4)	33 (16.3)
Geriatric education			
Before specialty - Received	20 (9.9)	20 (9.9)	40 (19.8)
Before specialty - Not received	103 (50.7)	60 (29.6)	163 (80.2)
After specialty - Received	9 (4.4)	5 (2.5)	14 (6.9)
After specialty - Not received	114 (56.2)	75 (36.9)	189 (93.1)
Age defined as the elderly patient			
65	37 (18.2)	32 (15.8)	69 (34.0)
70	47 (23.2)	25 (12.3)	72 (35.5)
Over 70	39 (19.2)	23 (11.3)	62 (30.5)
Total Participants			203 (100)

**Figure 1:**
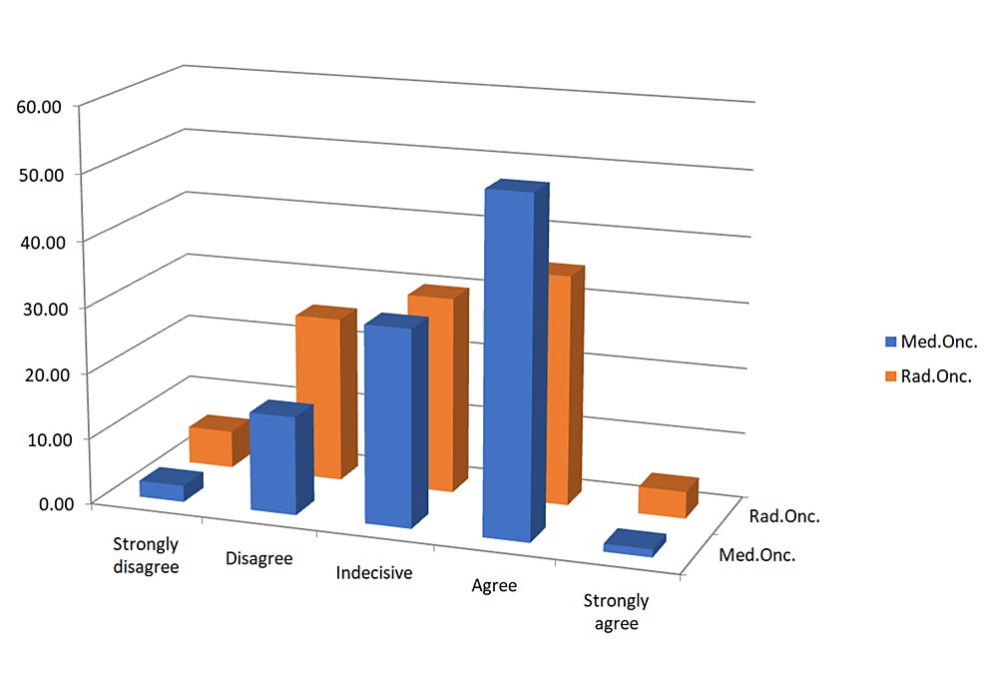
Percentage of physicians (medical oncologists and radiation oncologists) who think that they can provide relevant management in polypharmacy, falls, incontinence and multiple comorbid conditions observed in elderly people

The rate of receiving geriatric education before oncology education was 19.7% and this rate was 6.9% during oncology education. The rate of receiving education on topics such as delirium, mental capacity assessment, falls, polypharmacy, and urinary retention that may affect elderly patients was 31.2%. 1.5% of them stated that they received their training online and 5.9% from sessions in symposiums or congresses. However, 41% of the physicians think that they can provide relevant management in polypharmacy, falls, incontinence and multiple comorbid conditions observed in elderly people (Figure 1). The use of geriatric assessment tools in determining suitability for radiation therapy was rare. 88.6% of the radiation oncologists and 86.3% of medical oncologists rarely or never used it. Cognitive assessment was used regularly by only 58% of the participants. One hundred seventy-seven of the physicians (86.3%) thought that geriatric evaluation can independently predict overall survival rate. According to the physicians, patients' cognitive status, functional status, physiological age, polypharmacy, geriatric specialist recommendations, inpatient services, patient relatives and similar factors were found to be effective in deciding on treatment (Table 2). More than half of the physicians have followed up older patients more frequently than younger patients.

**Table 2:** Factors affecting the treatment decision in geriatric patients

Factors affecting the treatment decision in geriatric patients	Extremely Important	Important	Indecisive	Less Important
Chronological age	10 (4.9%)	122 (60.1%)	3 (1.5%)	61 (30.0%)
Cognitive status	45 (22.2%)	138 (68.0%)	5 (2.5%)	15 (7.4%)
Functional state	65 (32.0%)	125 (61.6%)		13 (6.4%)
History of fall	18 (8.9%)	145 (71.4%)	15 (7.4%)	23 (11.3%)
Social support state	24.1 (76.3%)	136 (67.0%)		17 (8.4%)
Comorbidity	84 (41.4%)	105 (51.7%)	2 (1.0%)	12 (5.9%)
Dietitian suggestions	22 (10.8%)	144 (70.9%)	14 (6.9%)	21 (10.3%)
Polypharmacy	38 (18.7%)	136 (67.0%)	11 (5.4%)	18 (8.8%)
Geriatric specialists' suggestions	27 (13.3%)	136 (67.0%)	24 (11.8%)	15 (7.4%)
Diet	64 (31.5%)	124 (61.1%)	3 (1.5%)	12 (5.9%)
Depression	44 (21.7%)	136 (67.0%)	7 (3.0%)	13 (6.4%)
Senior colleague recommendations	22 (10.8%)	154 (75.9%)	12 (5.9%)	14 (6.9%)
Physiological age	64 (31.5%)	121 (59.6%)	7 (3.4%)	11 (5.4%)
Patients' parents requests	17 (8.4%)	118 (58.1%)	22 (10.8%)	34 (16.4%)
Inpatient service facilities	40 (19.7%)	131 (64.5%)	14 (6.9%)	17 (8.4%)
Patients' biochemical findings	55 (27.1%)	124 (61.1%)	8 (3.9%)	15 (7.4%)

57.6% of the physicians were confident in the treatment of elderly cancer patients. When deciding whether to treat by radiotherapy or chemotherapy, only three (1.5%) physicians received support from geriatric specialists. 52.7% of the physicians indicated that more training about the management of elderly patients would be beneficial. 52.7% of physicians believed that specific learning objectives related to the management of the elderly with cancer patients would be valuable in the radiation oncology and medical oncology education curriculum. 51.7% of the physicians stated that geriatric evaluation may independently increase the survival rate in some oncological diseases and may affect the treatment regulation. In addition, 92.7% of them declared that getting an education would change their perspective on elderly patients. 69.8% of the physicians stated that they would prefer face-to-face training on the approach to geriatric patients (Table 3).

**Table 3:** Opinions regarding education on geriatric oncology

Opinions regarding education on geriatric oncology	Radiation Oncologists N (%)	Medical Oncologists N (%)	Total N (%)
Confident in the treatment	75 (62.5)	61 (76.3)	136 (67.0)
Need more training	116 (96.7)	72 (90.0)	188 (93.5)
Need specific learning objectives in the education curriculum	117 (97.5)	74 (92.6)	191 (94.1)
Prefer face-to-face training	82 (68.3)	57 (71.3)	139 (68.5)
Prefer online education	68 (56.7)	40 (50.0)	108 (53.2)
Believe education would change perspective	117 (97.5)	71 (88.8)	188 (92.6)
Total Participants	120 (100)	80 (100)	200 (100)

Factors affecting radiotherapy fraction dose selection for radiation oncologists and chemotherapy dose selection for medical oncologists were summarized in Table 4. Patient age, patient preference, social support, physiological age, patient's disease, access to hospital, accommodation, location of cancer, receiving concomitant treatment or not, cognitive function status, and opinion of senior colleagues were reported as important factors in both groups.

**Table 4:** Factors affecting radiotherapy fraction dose selection for radiation oncologists and chemotherapy dose selection for medical oncologists

Factor	Radiation Oncologists Important N (%)	Radiation Oncologists Not Important N (%)	Medical Oncologists Important N (%)	Medical Oncologists Not Important N (%)	Total Important N (%)	Total Not Important N (%)
Patients’ age	89 (72.4%)	33 (26.8%)	62 (77.6%)	18 (22.6%)	151 (74.4%)	51 (25.1%)
Patients’ choice	87 (70.8%)	30 (24.4%)	70 (87.6%)	9 (11.3%)	157 (78.5%)	39 (19.5%)
Social support	104 (84.5%)	17 (13.8%)	76 (95.1%)	3 (3.8%)	180 (89.1%)	20 (10.0%)
Physiological age	106 (86.2%)	14 (11.4%)	74 (92.6%)	4 (5.0%)	180 (89.1%)	18 (9.0%)
Patients’ disease	114 (92.7%)	8 (6.5%)	78 (97.5%)	2 (2.5%)	192 (95.0%)	10 (5.0%)
Convenient access, accommodation, and transportation	110 (89.4%)	13 (10.5%)	77 (96.3%)	3 (3.8%)	187 (93.5%)	16 (8.1%)
Using geriatric assessment	97 (78.9%)	26 (21.1%)	72 (90.0%)	4 (5.0%)	169 (84.2%)	30 (15.0%)
Anatomic localization	107 (87.0%)	13 (10.6%)	67 (83.8%)	8 (10.0%)	174 (85.8%)	21 (10.3%)
Concurrent systemic therapy	112 (91.1%)	10 (8.1%)	74 (92.6%)	4 (5.0%)	186 (91.8%)	14 (6.8%)
Cognitive status (Delirium, Dementia)	112 (91.1%)	9 (7.3%)	76 (95.0%)	3 (3.8%)	188 (93.0%)	12 (5.9%)
Opinion of senior colleagues or clinical supervisors	101 (81.1%)	15 (12.0%)	72 (90.0%)	5 (6.3%)	173 (85.6%)	20 (9.8%)

Thirty-five percent of radiation oncologists find comorbidities, 28.5%, functional status, 28.5% physiological age, 27.6% diet very important for decision of radiotherapy treatment, while 51.3% of medical oncologists think that comorbidities, 37.8% diet, 37.5% functional status, 36.3% physiological age very important for decision of systemic treatment. These findings were similar in both groups. Medical oncologists also indicate that they could monitor geriatric patients differently than younger patients (p=0.000028).

Trained physicians use the mini mental test to evaluate cognitive functions more frequently than non-trained physicians (p=0.02). The rate of consulting a geriatrician while treating the geriatric patient was found to be significantly higher in the trained group, compared to the untrained group (p=0.001). 53.1% of physicians with experience less than 10 years, and 77.6% of physicians with experience more than 10 years, defined patients aged 70 and over as geriatric patients (p=0.0003). Thirty-three physicians with experience exceeding 20 years have relatively less education about geriatric patients (p=0.04). Physicians with experience less than 10 years stated that the approach to geriatric patients will change with training, compared to those with experience over 10 years (p=0.02) and physicians with experience less than 20 years stated that the approach to geriatric patients will change with training (p=0.01).

## Discussion

To our knowledge, this is the first survey performed in order to evaluate the approach and education of both radiation and medical oncologists to geriatric cancer patients in Turkey. In many countries around the world, education programs have been added to the specialty training program for those who are going to treat the geriatric oncology patients, however geriatric oncology-specific formal training is not included in the curriculum for oncology education in our country. Unfortunately, clinicians who provide care for elderly patients worldwide receive little or no formal training, hence, due to lack of adequate knowledge on the subject, distrust is observed in physicians [10]. The study conducted by Leifer et al. [11], which investigated the interest and training of radiation oncologists in geriatric oncology in Canada reported that the knowledge of 83% of physicians was found to be insufficient in this regard, and similarly, in the study conducted by Maggiore et al. [12] among hematology specialists, the knowledge of the physicians in treating geriatric oncology patients was found to be insufficient.

As a result of our survey, 93.2% of the physicians did not receive any training during their oncology training. Also, 16.8% of radiation oncologists and 25% of medical oncologists received geriatric training before their oncology training. Only 5.9% of them received formal training given by geriatricians and geriatric oncology specialists. Studies have shown that performance status, survival benefit of treatment, life expectancy and quality of life were factors affecting the treatment decision, and similar answers were stated by the oncologists in our study. According to the physicians, patients' cognitive status, functional status, physiological age, polypharmacy, geriatric specialist recommendations, inpatient services and patient relatives’ requests also affected the decision of the physicians regarding treatment [13-15] similar to our findings. Different than other studies, we had the chance to compare the factors affecting the treatment decision in geriatric patients between radiation oncologists and medical oncologists. We observed that factors affecting the treatment decision were similar in both groups. In addition, the factors affecting the radiotherapy fraction dose and chemotherapy dose selection were similar in our study and these are comorbidities, diet, functional status and physiological age. When we compare our study with the other studies, social support is also effective in treatment-type decisions.

Comprehensive Geriatric Assessment (CGA) is the “gold standard” for evaluating older adults. The origin of CGA dates back to the 1940s and was developed by Dr. Marjory Warren from the UK when she needed better hospital evaluation of elderly patients who were bedridden due to chronic diseases [16]. Over time, it has been accepted in many countries and geriatric centers. Today, many screening tools, forms and questionnaires including the experiences of different centers are used. G8 screening questionnaire, The Vulnerable Elders Survey-(VES-13), Flemish version of the triage risk screening tool (TRST), Osteoporotic fracture index, Groningen frailty indicator, Fried’s frailty criteria, Abbreviated comprehensive geriatric assessment (aCGA) test are the most commonly used of these tests and screening tools [17-19]. Generally, the rate of usage of the geriatric assessment scales is low (10.7%) for determining treatment suitability in geriatric patients. In our study, it was stated that cognitive status was taken into account when making a treatment decision, however mini mental tests were used only 11.7%. In most cases, the evaluation of the geriatric patients and the subsequent treatment decision-making were performed without any testing.

In the surveys conducted up to date, it has been observed that oncologists refrain from intensive cancer treatment in the elderly patient group while giving treatment. For this reason, many patients who otherwise would benefit treatment may remain untreated due to their chronological age. Ulger et al. stated that considering only chronological age as the decision factor is insufficient to guide treatment and survival will increase if geriatric patients who can tolerate aggressive treatment are distinguished and such group of patients will not be left untreated [20].

Loh et al. underlined that geriatric assessment can identify areas of vulnerability, predict survival and toxicity, help in clinical treatment decisions, and guide interventions in routine oncology practice. Ideally, all older patients who are being considered for cancer treatments should receive a geriatric assessment as a part of their evaluation; however, in settings of limited time and resources, a geriatric screening tool could be used [21]. Shahrokni et al. detected that keeping a patient-centered mindset and enabling older patients to be involved in choices are crucial to providing effective and efficient care. Communication among members of the care team is the key to avoiding fragmented care, unnecessary expense, and unwanted outcomes. Targeted interventions could result in significant improvements in the quality of life of older patients with cancer throughout the cancer continuum [22].

Physicians in our study did not feel confident in deciding the treatment on geriatric oncology patients. 57.6% of the radiation oncologists and medical oncologists stated that additional education during their training for elderly patients could change their perspectives and addition of specific learning objectives about geriatric oncology in education curriculum is needed. Younger physicians are more inclined to receive training on the management of geriatric patients and 69.8% of them prefer to have face-to-face training, while the rate of those who want online education is 53.2%. Such training is important not only for the oncologists but also for all other disciplines dealing with the geriatric patient group on geriatric principles as well. Even an online education can be very effective for the education of a large audience and can be a stepping stone for further education.

## Conclusions

In conclusion, the geriatric cancer patient population is a heterogeneous group. Comprehensive evaluation, individualization of the treatment decision and proper management will increase the quality of life and survival of geriatric cancer patients. We conclude that the formal education about geriatric oncology in Turkey appears to be inadequate. Improvement of geriatric-based competencies within oncology education “core curriculum” is needed. Our results provide perspectives for developing knowledge and skills in geriatric training among radiation and medical oncologists, so that oncologists who will confront more geriatric cancer patients in line with the increase in the elderly population will feel and act more confident in providing appropriate cancer treatments and care to the elderly patients.

## Appendices

**Table 5:** STROBE Statement Checklist of items that should be included in reports of observational studies

Section/Topic	Item No	Recommendation	Reported on Page No
Title and abstract	1	(a) Indicate the study’s design with a commonly used term in the title or the abstract	1
		(b) Provide in the abstract an informative and balanced summary of what was done and what was found	1-2
Introduction			
Background/rationale	2	Explain the scientific background and rationale for the investigation being reported	3
Objectives	3	State specific objectives, including any prespecified hypotheses	3-4
Methods			
Study design	4	Present key elements of study design early in the paper	4
Setting	5	Describe the setting, locations, and relevant dates, including periods of recruitment, exposure, follow-up, and data collection	4
Participants	6	(a) Cohort study—Give the eligibility criteria, and the sources and methods of selection of participants. Describe methods of follow-up Case-control study—Give the eligibility criteria, and the sources and methods of case ascertainment and control selection. Give the rationale for the choice of cases and controls Cross-sectional study—Give the eligibility criteria, and the sources and methods of selection of participants	4
		(b) Cohort study—For matched studies, give matching criteria and number of exposed and unexposed Case-control study—For matched studies, give matching criteria and the number of controls per case	4
Variables	7	Clearly define all outcomes, exposures, predictors, potential confounders, and effect modifiers. Give diagnostic criteria, if applicable	4
Data sources/measurement	8*	For each variable of interest, give sources of data and details of methods of assessment (measurement). Describe comparability of assessment methods if there is more than one group	4
Bias	9	Describe any efforts to address potential sources of bias	-
Study size	10	Explain how the study size was arrived at	-
Quantitative variables	11	Explain how quantitative variables were handled in the analyses. If applicable, describe which groupings were chosen and why	-
Statistical methods	12	(a) Describe all statistical methods, including those used to control for confounding	4
		(b) Describe any methods used to examine subgroups and interactions	4
		(c) Explain how missing data were addressed	-
		(d) Cohort study—If applicable, explain how loss to follow-up was addressed Case-control study—If applicable, explain how matching of cases and controls was addressed Cross-sectional study—If applicable, describe analytical methods taking account of sampling strategy	4
		(e) Describe any sensitivity analyses	-

Section/Topic	Item No	Recommendation	Reported on Page No
Results			
Participants	13*	(a) Report numbers of individuals at each stage of study—eg numbers potentially eligible, examined for eligibility, confirmed eligible, included in the study, completing follow-up, and analysed	4-5
		(b) Give reasons for non-participation at each stage	-
		(c) Consider use of a flow diagram	-
Descriptive data	14*	(a) Give characteristics of study participants (eg demographic, clinical, social) and information on exposures and potential confounders	4-5
		(b) Indicate number of participants with missing data for each variable of interest	-
		(c) Cohort study—Summarise follow-up time (eg, average and total amount)	4
Outcome data	15*	Cohort study—Report numbers of outcome events or summary measures over time	4
		Case-control study—Report numbers in each exposure category, or summary measures of exposure	5
		Cross-sectional study—Report numbers of outcome events or summary measures	5
Main results	16	(a) Give unadjusted estimates and, if applicable, confounder-adjusted estimates and their precision (eg, 95% confidence interval). Make clear which confounders were adjusted for and why they were included	4-6
		(b) Report category boundaries when continuous variables were categorized	4-6
		(c) If relevant, consider translating estimates of relative risk into absolute risk for a meaningful time period	4-6
Other analyses	17	Report other analyses done—eg analyses of subgroups and interactions, and sensitivity analyses	5-6
Discussion			
Key results	18	Summarise key results with reference to study objectives	6
Limitations	19	Discuss limitations of the study, taking into account sources of potential bias or imprecision. Discuss both direction and magnitude of any potential bias	7-8
Interpretation	20	Give a cautious overall interpretation of results considering objectives, limitations, multiplicity of analyses, results from similar studies, and other relevant evidence	7
Generalisability	21	Discuss the generalisability (external validity) of the study results	7-8
Other Information			
Funding	22	Give the source of funding and the role of the funders for the present study and, if applicable, for the original study on which the present article is based	1
